# Placement, management and complications associated with peripheral intravenous catheter use in UK small animal practice

**DOI:** 10.1111/jsap.13782

**Published:** 2024-09-05

**Authors:** E. Haskey, V. Maund, F. Allerton, B. Browse, C. Heard, C. O'Donnell, K. Davison, C. Hertel, E. Booth, S. Lawrence, E. Dever, L. Bowe, H. Taylor, K. Hall, K. Trimble, M. Junior, C. Fennell, N. Stevenson, A. Sterritt, E. Penn, L. Nowell, A. Collins, E. Jones, C. Scudder

**Affiliations:** ^1^ Clinical Sciences and Services Royal Veterinary College Potters Bar UK; ^2^ Willows Veterinary Centre and Referral Service Solihull UK; ^3^ Cave Veterinary Specialists Wellington UK; ^4^ Highcroft Veterinary Specialists Bristol UK; ^5^ Paragon Veterinary Referrals Wakefield UK; ^6^ Quarry Veterinary Group Shrewsbury UK; ^7^ Davies Veterinary Specialists Hertfordshire UK; ^8^ Kentdale Referrals Milnthorpe UK; ^9^ Dick White Referrals Cambridgeshire UK; ^10^ Wear Referrals Veterinary Specialist & Emergency Hospital Stockton‐on‐Tees UK; ^11^ Southfields Veterinary Specialists Essex UK; ^12^ Garston Veterinary Group Somerset UK; ^13^ Taverham Veterinary Hospital Norwich UK; ^14^ Willows Veterinary Group Cheshire UK; ^15^ Station House Vets York UK; ^16^ Village Vets Ellesmere Port UK; ^17^ North Wales Veterinary Referrals Buckley UK

## Abstract

**Objectives:**

To describe the techniques for preparation and placement of peripheral intravenous catheters (PIVCs), to describe the complications associated with PIVCs, and to identify factors associated with PIVC complications in small animal practice in the United Kingdom.

**Materials and Methods:**

A prospective multicentre observational study was undertaken between January 2022 and January 2023. Data collected included patient information, information regarding the placement and maintenance of PIVCs, and PIVC complications, from privately owned cats and dogs presenting to veterinary institutes in the United Kingdom. Patients required a PIVC to be placed as part of their care and the PIVC was anticipated to be in situ for >24 hours to be eligible for PIVC complication analysis.

**Results:**

A total of 19 institutes recorded data regarding 382 PIVCs, with 325 (85.1%) placed in dogs and 57 (14.9%) in cats. The most common reasons for placement were to administer intravenous fluid therapy (74.3%) and intravenous medications (71.7%). There were 102 of 382 (26.7%) PIVCs associated with a complication, with limb swelling/suspected phlebitis in 44 of 382 (11.5%) and PIVC dislodgement/patient interference in 30 of 382 (7.9%) PIVCs. Factors associated with increased risk of complication were more than 1 attempt to place the PIVC, a second or subsequent PIVC being placed during hospitalisation, flush frequency different than every 1 to 24 hours, and flush solution with compound sodium lactate.

**Clinical Significance:**

Veterinary professionals must be vigilant when monitoring a patient with a PIVC in situ, particularly if a PIVC is associated with one of the aforementioned factors of increased likelihood of complication.

## INTRODUCTION

Peripheral intravenous catheters (PIVCs) can be utilised to administer a range of treatments such as intravenous fluid therapy, medication and blood products. PIVCs are currently the most commonly placed invasive device in human medicine, with over 2 billion PIVCs purchased per year worldwide (Keogh et al., 2020). PIVCs are associated with numerous complications which include infectious complications, such as bacteraemia or local infections, and non‐infectious complications including dislodgement, occlusion, extravasation, phlebitis and dermatitis either induced by skin antisepsis or bandaging materials (Liu et al., 2020; Mimoz et al., 2015). The prevalence of PIVC complications in human medicine varies between 20 and 40 per 100 PIVCs, with complications increasing the risk of death, adding to patient treatment costs and delaying time to discharge (Marsh, Webster, Larsen, Cooke, et al., 2018). The proportion of hospitalised cats and dogs that have PIVCs inserted in veterinary practice is unknown, but in the authors' experience almost all cats and dogs hospitalised for >24 hours will have a PIVC in situ. Between 17% and 47% of PIVCs placed in cats and dogs have been associated with a complication, with phlebitis, extravasation, PIVC dislodgement and occlusion being the most common (Bush et al., 2020; Crisi et al., 2022; Simpson & Zersen, 2022, 2023). Patient welfare is compromised, and the cost of patient care is increased as a consequence of these complications.

Factors that have been associated with increased risk of a PIVC complication in veterinary medicine include increasing length of hospitalisation, increasing weight (dogs only), personnel placing the device and lack of use of a force‐activated separation device (Granger et al., 2023; Guzmán Ramos et al., 2018; Parkes, 2015; Simpson & Zersen, 2022, 2023). Whilst there are guidelines published by the American Animal Hospital Association for PIVC placement, there are no consensus statements describing best practice for PIVC placement and management in veterinary medicine (AAHA Guidelines, 2021). A better understanding of the factors associated with PIVC complications could allow for the development of a veterinary evidence‐based PIVC care bundle. Similar care bundles have been implemented in human medicine and have resulted in decreased PIVC complications, increased indwell times and there has been a trend to lower PIVC‐related infections (Boyd et al., 2011; DeVries et al., 2016; Kleidon et al., 2019). An effective care bundle could therefore result in decreased patient morbidity and decreased costs associated with PIVC use.

The aims of this study were to describe patient and personnel preparation for PIVC placement, to describe complications of PIVCs and to identify factors associated with PIVC complications. We hypothesised that patient factors, including PIVC being placed in a thoracic limb, requirement of only one PIVC during hospitalisation, calm patient demeanour and personnel factors, including use of hand hygiene prior to placement, and placement by a registered veterinary nurse (RVN) would be associated with a decreased likelihood of a PIVC complication. We also hypothesised that frequency of PIVC flushing every 1 to 4 hours would decrease the likelihood of a PIVC complication compared to less frequent flushing, PIVC flush solution type would not influence the likelihood of a complication and administration of steroid medications or known irritant medications into the PIVC would increase the likelihood of a PIVC complication.

## MATERIALS AND METHODS

### Study design and inclusion criteria

This was a multicentre, prospective, observational study undertaken in UK primary care and referral veterinary institutes between January 2022 and January 2023. Ethical approval was obtained prior to data collection (blinded for review). Institutes were recruited by advertising the study at veterinary conferences, an online veterinary forum (vetsurgeon.org) and an article written in a UK veterinary news publication (vettimes.co.uk). Privately owned cats and dogs presenting to veterinary institutes who underwent PIVC placement as part of their care were evaluated for inclusion. Data from only one PIVC per patient was included in this study. This may have included the first PIVC placed or a subsequent PIVC placed during hospitalisation (i.e., second or third PIVC placed). PIVCs were included if they were intended to be in situ for at least 24 hours at the time the PIVC was placed. We believed PIVCs intended to be in situ for <24 hours may not be placed or bandaged in the same manner as those PIVCs intended for longer‐term use. Patients were excluded if they were transferred to another institute while their PIVC was in place due to concern patients travelling between institutes may experience different PIVC management techniques, or if the PIVC was used to administer chemotherapy as those patients may have their PIVCs placed in a different manner because of the requirement for a clean first stick and the administration of chemotherapy itself may increase the risk of vasculitis. Each institute was asked to collect data for 3 weeks, and preferentially for these weeks to not be consecutive weeks.

### Data recording

Data was initially collected on a paper questionnaire containing 43 questions (Data S1) and then uploaded onto an online electronic GDPR‐compliant, data platform (Castor Electronic Data Capture). Each institute had up to two members of their team (either RVN or veterinarians) nominated to aid design of the paper questionnaires and undertake the data collection. Online meetings determined the factors included in the questionnaire, the methodology of data collection and to achieve uniformity of the recording of PIVC complications.

The data captured were separated into patient data, PIVC placement and maintenance, and PIVC removal. The patient data section included the date of PIVC placement, hospital ID number, species, breed, gender, neuter status and the primary patient body system affected (if the primary reason for presentation could not be defined then the body system was classed as “other”). The PIVC placement and maintenance section included the reason for PIVC insertion, the role of the individual placing the PIVC being a veterinarian, veterinary nurse or student (either veterinarian student or veterinary nurse student), recording of hand hygiene performed prior to placement (as defined by either washing hands or applying alcohol sanitiser, or both) and whether non‐sterile or sterile gloves were worm, whether the appointment was for routine or emergency reasons (an emergency appointment was defined as a condition which the veterinary team determined could not wait for >24 hours to be treated), patient mentation (reported as conscious, sedated or under general anaesthesia) demeanour (recorded as calm, restless, showing signs of aggression, unable to place the PIVC without chemical restraint or “other”), location of patient restraint for PIVC placement (either on the floor, table or “other”), number of attempts needed to place the PIVC in that vein, PIVC number in situ after placement of the PIVC, and whether it was the first PIVC placed during hospitalisation or a subsequent one. Details regarding patient skin preparation which were recorded included whether hair was clipped or shaved, was the amount of hair removed deemed appropriate, the disinfectants used on the skin, what materials were used to apply the disinfectants, was a local anaesthetic applied to the skin, the PIVC brand and size, vein of PIVC placement, whether a facilitative incision was used, whether extension sets were attached to the PIVC, and the tape material used to secure the PIVC to the skin. Data regarding the use of the PIVC included whether blood samples were collected via the PIVC, whether the PIVC was flushed and if so, how frequently and if there was no consistent time interval between flushing then they were recorded as “other,” was a fluid bolus administered in the PIVC (fluid bolus was defined as ≥10 mL/kg fluid administered within 20 minutes), and the medications administered in the PIVC. The medication types were grouped into antibiotics (which included amoxicillin‐clavulanate, cefuroxime, marbofloxacin and metronidazole), anti‐emetics (which included metoclopramide, maropitant and ondansetron), glucocorticoids (which included dexamethasone sodium phosphate, hydrocortisone sodium succinate and methylprednisolone sodium succinate), packed red blood cells, whole blood, fresh frozen plasma, frozen plasma or other. When a patient underwent CT imaging then whether a pressure injector used to administer a contrast agent was recorded. With regards to hygiene of PIVC management, the following data was recorded; whether the drip line was disconnected from the PIVC if a dog was taken outside, whether chlorhexidine disinfection caps were used if the drip line was disconnected and whether injection ports were disinfected prior to use and if so, with what disinfectant. Within the PIVC removal section, the date of PIVC removal was recorded, the time the PIVC was in situ was recorded, whether the PIVC was removed prematurely (prematurely was defined as earlier than that institute would routinely remove PIVCs in their patients), and PIVC complications were recorded. PIVC complications were categorised as “discharge originating from the PIVC site,” “dislodgement/patient interference,” “limb swelling/suspected phlebitis,” “occlusion” and “soiled bandaging.” Dislodgement was defined as the movement of PIVC resulting in the hub no longer being immediately adjacent to the insertion site. Patient interference was defined as the patient interfering with and damaging the PIVC and/or extension set. Limb swelling was defined as engorgement of the soft tissues of the limb either immediately surrounding the PIVC site or distal to it. Occlusion was defined as blockage of the lumen resulting in increased resistance or inability to PIVC flush or administer medication. Soiled bandaging was defined as bandaging that had become moist or contaminated with bodily fluid or dirt to the point of requiring replacement. An assessment of the limb at the time of PIVC removal was to be performed for evidence of phlebitis, as defined by the presence of erythema or pain, and a phlebitis score was assigned using the “Ward Phlebitis Score Chart 2018” which attributes scores from 0 to 4 utilising the limb colour, temperature, swelling and evidence of pain at the PIVC site (Hancill & Ward, 2019). Whether the PIVC tip underwent bacterial culture and any bacteria cultured were recorded (for the full list of data recorded see Data S1).

### Statistical analysis

Statistical analysis was performed using commercially available software (IBM SPSS Statistics version 29, IBM Corporation, New York, NY). Data are presented as number of events and percentage. A chi‐squared test was used to compare the proportion of PIVC complications between institutes. Where appropriate based on clinical relevance, variables were assigned into new groups of a minimum size of 10% of the dataset. For example, the number of attempts to place the PIVC was recategorised into 1 attempt and >1 attempt. Factors associated with PIVC complication were initially assessed using a univariable binary regression model, and factors with a P‐value <0.1 were used in a backwards inclusion multivariate binary regression analysis. Significance was determined as a P‐value <0.05. PIVCs, where a complication (presence or absence) was not reported, were excluded from the complication analyses. Missing data were omitted from analyses.

## RESULTS

The complete dataset is available for review (PIVCA dataset, 2023).

### Institutes and patient inclusion

There were 19 institutes that uploaded data for 382 PIVCs; 14 of 382 (3.7%) PIVCs were placed in mixed practice and 368 of 382 (96.3%) PIVCs were placed in small animal practices, with 335 of 382 (87.7%) PIVCs placed in 17 institutes with Royal College of Veterinary Surgeons (RCVS) hospital status. A summary of the number of PIVCs submitted per institute is presented in Table 1. The patient sex, neuter status, most common breeds, presentation as routine versus emergency, primary presenting condition by body system and medications administered in the PIVC are presented in Table 2.

**Table 1:** A summary of the number of peripheral intravenous catheter (PIVC) submission per institute

Number of PIVCs submitted per institute	Number of institutes
61 to 70	1
51 to 60	1
41 to 50	2
31 to 40	2
21 to 30	0
11 to 20	4
0 to 10	8

**Table 2:** Signalment, reason for presentation and medications administered in 382 peripheral intravenous catheter

Variable	Dogs (*n* = 325), *n* (%)	Cats (*n* = 57), *n* (%)
Sex and neuter status		
Male neutered	109 (33.5)	30 (52.6)
Male entire	55 (16.9)	2 (3.5)
Male unknown neuter status	7 (2.2)	1 (1.8)
Female neutered	106 (32.6)	22 (38.6)
Female entire	44 (13.5)	2 (3.5)
Female unknown neuter status	4 (1.2)	0
Most common breeds	Cross‐breed = 63 (19.4)	Domestic short hair = 33 (57.9)
	Labrador = 44 (13.5)	Domestic long hair = 4 (7.0)
	dachshund = 22 (6.8)	British short hair = 3 (5.3)
	French bulldog = 21 (6.5)	Siamese = 3 (5.3)
Presentation type		
Routine	178 (54.8)	17 (29.8)
Emergency	143 (44.0)	40 (70.2)
Data missing	4 (1.2)	0
Primary presenting condition by body system		
Cardiovascular	20 (6.2)	2 (3.5)
Dermatological	7 (2.2)	0
Endocrine	15 (4.6)	4 (7.0)
Gastrointestinal	37 (11.4)	9 (15.8)
Hepatobiliary and pancreas	14 (3.7)	2 (3.5)
Immune‐mediated / haematological	15 (4.6)	2 (3.5)
Neurological	54 (16.6)	4 (7.0)
Orthopaedic	45 (13.8)	3 (5.3)
Other	38 (11.7)	10 (17.5)
Respiratory	23 (7.1)	7 (12.3)
Trauma	2 (0.6)	4 (7.0)
Urological/reproductive	13 (4.0)	8 (14.0)
Missing	42 (12.9)	2 (3.5)
Medications administered		
Intravenous fluid therapy	246 (75.7)	38 (66.7)
Other intravenous medication	242 (74.5)	32 (56.1)
General anaesthesia/sedation	234 (72.0)	25 (43.9)
Other	8 (2.5)	6 (10.5)
Missing	0	0

### PIVC placement

Results pertaining to preparation and placement of the PIVC are presented in Table 3. Hand hygiene was performed prior to placement of 222 of 382 (58.1%) PIVCs. Hand hygiene was performed by 19 of 23 (82.6%) students (either veterinary nurse or veterinarian student), 161 of 271 (59.4%) RVNs and 42 of 83 (51.2%) veterinarians. Gloves were worn for the placement of 64 of 382 (16.8%) PIVCs, with non‐sterile clinical gloves worn for 63 of 64 (98.4%) and sterile clinical gloves worn for one of 64 (1.6%). Patient demeanour was reported to be calm and relaxed for 228 of 382 (59.7%), agitated or nervous for 102 of 382 (26.7%), exhibiting signs of aggressive behaviour for 16 of 382 (4.2%), unsuitable for PIVC placement without sedation for 24 of 382 (6.3%), obtunded for one of 382 PIVC placement and not recorded for 11 of 382 (2.9%) PIVCs. The PIVC was placed successfully on the first attempt for 311 of 382 (81.4%), on the second attempt for 52 of 382 (13.6%) and on the third or more attempts for 15 of 382 (3.9%) PIVCs and was missing for four PIVCs.

**Table 3:** Summary of the results describing placement and time in situ of 382 peripheral intravenous catheter (PIVC)

Variable	Categories
Personnel placing the PIVC	Veterinary nurse = 271 (70.9%) Veterinarian = 83 (21.7%) Student_†_ = 23 (6%) Missing data = 5 (1.3%)
Hand hygiene prior to PIVC placement	Alcohol sanitiser only = 124 (32.5%) Hand washing only = 75 (19.6%) Hand washing and alcohol sanitiser = 23 (6.0%) Not performed = 125 (32.7%) Missing data = 35 (9.2%)
Location patient was restrained for placement	Dogs Table = 143 (44.0%) Floor = 170 (52.3%) In someone's arms = 1 (0.3%) Missing = 11 (3.4%) Cats Table = 56 (98.2%) Floor = 1 (1.8%) Missing data = 0
Patient skin disinfectant	Alcohol only = 71 (18.6%) Alcohol and <2% chlorhexidine = 58 (15.2%) Alcohol and 2% chlorhexidine = 180 (47.1%) Alcohol and >2% chlorhexidine = 68 (17.8%) Missing data = 5 (1.3%)
Topical local anaesthetics applied prior to placement	EMLA 5% cream^‡^ = 39 (10.2%) EthyCalm spray^§^ = 26 (6.8%) Lidocaine liquid (unknown percentage) = 4 (1.0%) Intubeaze® 20 mg/mL spray^¶^ = 1 (0.3%) None = 284 Missing = 28
PIVC vein location	Dog Auricular vein = 5 (1.5%) Cephalic (accessory or branch) vein = 24 (7.4%) Cephalic (main) vein = 269 (82.8%) Dorsal pedal vein = 4 (1.2%) Saphenous (lateral) vein = 17 (5.2%) Missing data = 6 (1.8%) Cat Cephalic (accessory or branch) vein = 2 (3.5%) Cephalic (main) vein = 51 (89.5%) Saphenous (medial) vein = 4 (7.0%) Missing data = 0
PIVC material	Dog Teflon = 252 (71.6%) Polyurethane = 67 (20.6%) Missing = 6 (1.8%) Cats Teflon = 42 (73.7%) Polyurethane = 15 (26.3%)
PIVC size	Dog 18 gauge = 15 (4.6%) 20 gauge = 182 (56.0%) 22 gauge = 114 (35.0%) 24 gauge = 7 (2.2%) Missing data = 7 (2.2%) Cat 20 gauge = 1 (1.8%) 22 gauge = 53 (93.0%) 24 gauge = 3 (5.2%) Missing data = 0
Consumables directly attached to the PIVC	Dog T‐port = 192 (59.1%) Y‐connector = 104 (32.0%) Intravenous giving set = 17 (5.2%) Bung = 5 (1.5%) Missing data = 7 (2.2%) Cat T‐port = 33 (57.9%) Y‐connector = 10 (17.5%) Intravenous giving set = 13 (22.8%) Bung = 1 (1.8%)
Needle free system used	Dog No = 38 (11.7%) Yes = 265 (81.5%) Missing data = 22 (6.8%) Cat No = 18 (31.6%) Yes = 36 (63.2%) Missing data = 3 (5.3%)
PIVC number since admission	Dog First = 247 (76.0%) Second = 58 (17.8%) Third = 9 (2.8%) Fourth = 4 (1.2%) Fifth = 2 (0.6%) Sixth = 1 (0.3%) Missing = 4 (1.2%) Cat First = 46 (80.7%) Second = 9 (15.8%) Third = 2 (3.5%) Missing = 0
Was the area of hair removal prior to PIVC deemed appropriate to allow sterile PIVC placement and correct application of skin tape?	Dog No = 5 (1.5%) Yes = 290 (89.2%) Missing data = 30 (9.2%) Cat No = 2 (3.5%) Yes = 53 (93.0%) Missing data = 2 (3.5%)
Bandaging material used to secure the PIVC in situ	Dog Duropore™^$^ = 121 (37.2%) Brown elastic adhesive bandage = 169 (52.0%) Other = 28 (8.6%) Missing = 7 (2.2%) Cat Duropore™^$^ = 24 (42.1%) Brown elastic adhesive bandage = 18 (31.6%) Other = 15 (6.3%)
Time PIVC was in situ	Dog 0 to 24 hours = 79 (24.3%) >24 to 48 hours = 125 (38.5%) >48 to 72 hours = 67 (20.6%) >72 to 96 hours = 29 (8.9%) >96 hours = 8 (2.5%) Missing data = 17 (5.2%) Cat 0 to 24 hours = 9 (15.8%) >24 to 48 hours = 20 (35.1%) >48 to 72 hours = 11 (19.3%) >72 to 96 hours = 11 (19.3%) >96 hours = 5 (8.8%) Missing data = 1 (1.8%)

^†^ Student refers to either a veterinary nurse student or veterinarian student

^‡^ EMLA cream 5% (Aspen, Dublin, Ireland)

^§^ EthyCalm spray (Invicta, Sussex, UK)

^¶^ Intubeaze® 20 mg/mL spray (Dechra, Shrewsbury, UK)

^$^ Duropore™, 3M™ Duropore™ Surgical Tape, Berkshire, UK

### PIVC maintenance and use

The results regarding the maintenance of the extension sets attached to the PIVC are presented in Table S1. There were consistent time intervals between PIVC flushing for 318 of 382 (83.2%) PIVCs, and 11 PIVCs were not flushed at set time intervals and grouped as “other” flushing frequencies. This group contained six of 382 (1.5%) PIVCs that were not flushed because the patient was receiving intravenous fluid therapy, three of 382 (0.8%) that had an inconsistent interval between flushings, one of 382 (0.3%) because flushing occurred only at the time of medication administration, and one of 382 (0.3%) which was reported to be flushed “as needed.” Data regarding flushing was missing for 53 of 382 (13.6%) PIVCs. The most common flush frequencies were every 4 hours (166/318, 52.2%), every 6 hours (70/318, 22.0%) and every 12 hours (61/318, 19.2%). The solution used for PIVC flushing was 0.9% sodium chloride for 303 of 382 (79.3%), 0.9% sodium chloride with heparin for 22 of 382 (5.8%), compound sodium lactate for 10 of 382 (2.6%) PIVCs, and information was not recorded for 47 of 382 (12.3%) PIVCs. There were 215 of 325 (66.2%) PIVCs in dogs and 30 of 57 (52.6%) PIVCs in cats that had intravenous fluid administered in them and were also flushed at consistent time intervals. Analgesia (non‐steroidal or opioid) was administered in 272 of 382 (71.2%) PIVCs, antibiotics in 156 of 382 (40.8%) PIVCs, anti‐emetic medications in 99 of 382 (25.9%) PIVCs and steroids in 17 of 382 (4.4%) PIVCs, and these data were missing for four PIVCs.

### PIVC complications

The PIVC complications are presented in Table 4. Complications that resulted in earlier than intended PIVC removal occurred in 68 of 325 (20.9%) PIVCs in dogs and nine of 57 (15.8%) PIVCs in cats. An additional 19 of 325 (5.8%) PIVCs in dogs and six of 57 (10.5%) PIVCs in cats had a complication identified at the time of intended PIVC removal. Therefore, the total number of PIVCs complications was 102 of 382 (26.7%). The most common complications were limb swelling/suspected phlebitis which occurred in 36 of 325 (11.1%) dogs and eight of 57 (14.0%) cats, and PIVC dislodgement/patient interference which occurred in 26 of 325 (8.0%) dogs and four of 57 (7.0%) cats.

**Table 4:** Peripheral intravenous catheter (PIVC) complications and phlebitis scores according to species

Variable	Dog, *n* (%)	Cat, *n* (%)
Complication resulting in premature PIVC removal	*n* = 325	*n* = 57
Discharge originating from PIVC site	1 (0.3)	0
Dislodgement and/or patient interference with PIVC	25 (7.7)	4 (7.0)
Limb swelling and/or suspected phlebitis	21 (6.5)	3 (5.3)
PIVC occlusion	11 (3.4)	1 (1.8)
Soiled bandaging	10 (3.1)	1 (1.8)
No complication identified resulting in premature PIVC removal	257 (79.1)	48 (84.2)
Missing	0	0
New complication identified at intended time of PIVC removal	*n* = 325	*n* = 57
Discharge originating from PIVC site	0	0
Dislodgement and/or patient interference with PIVC	1 (0.3)	0
Limb swelling and/or suspected phlebitis	15 (4.6)	5 (8.8)
PIVC occlusion	0	1 (1.8)
Soiled bandaging	3 (0.9)	0
No new complication identified at intended time of PIVC removal	306 (94.2)	51 (89.4)
Missing	0	0
Phlebitis scores of PIVCs prematurely removed	*n* = 68	*n* = 9
Score 0	41 (60.3)	6 (66.7)
Score 1	15 (22.1)	3 (33.3)
Score 2	9 (13.2)	0
Score 3	3 (4.4)	0
Missing data = 0	0	0
Phlebitis scores of PIVCs with complication identified at time of removal	*n* = 19	*n* = 6
Score 0	5 (27.8)	2 (33.3)
Score 1	8 (44.4)	2 (33.3)
Score 2	5 (27.8)	2 (33.3)
Score 3	0	0
Score 4	1 (5.6)	0
Missing data	0	0
Phlebitis scores of PIVCs recorded as having no complication	*n* = 238	*n* = 42
Score 0	199 (83.3)	36 (81.8)
Score 1	21 (8.8)	4 (9.1)
Missing data	18 (7.5)	2 (4.5)

Where both personnel role and complication outcome were reported, complications occurred in 75 of 259 (29.0%) PIVCs placed by RVNs, 25 of 83 (30.1%) PIVCs placed by veterinarians and two of 21 (9.5%) placed by students. The Ward Phlebitis Scores at the time of PIVC removal are presented in Table 4. There were 245 of 325 (75.4%) PIVCs in dogs which had a phlebitis score of 0, 44 of 325 (13.5%) with a score of 1, 14 of 325 (4.3%) with a score of 2, three of 325 (0.9%) with a score of 3, one of 325 (0.3%) with a score of 4 and the score was not recorded for 18 PIVCs. For the PIVCs in cats, 44 of 57 (77.2%) had a phlebitis score of 0, nine of 57 (15.8%) had a score of 1, two of 57 (3.5%) had a score of 2 and the score was not recorded for two PIVCs.

Only one of 382 PIVC was sent for bacterial culture. This PIVC was removed prematurely from a male cross‐breed dog whose reason for removal was “patient interference” and was associated with a phlebitis score of 0. No bacteria were cultured.

There were 19 PIVCs that had missing data regarding any possible complication. There was no difference in proportions of complications between institutes (χ^2^ (17, N = 363) = 15.9, P = 0.53). Univariable binary logistic regression analyses are presented in Table 5. There were four factors associated with increased odds of a PIVC complication with a P‐value of <0.1 which were then used in the multivariable regression analysis; number of attempts to place the PIVC >1 (OR 2.4; 95% CI 1.4 to 4.1, P = 0.003), being a second or subsequent PIVC (OR 2.5; 95% CI 1.5 to 4.3, P < 0.001), flush frequency (P = 0.03) with flushing every 12 to 24 hours having increased odds of 1.9 (95% CI 1.0 to 3.5, P = 0.05) and flushing “other” having increased odds of 6.8 (95% CI 1.6 to 28.3, P = 0.009) compared with flushing every 1 to 4 hours, and flush solution type (P = 0.02) with flushing with compound sodium lactate solution having increased odds of 6.7 (95% CI 1.7 to 26.4, P = 0.007) compared to 0.9% sodium chloride. The results of the multivariable analyses are presented in Table 6. More than one attempt to place the PIVC (OR 2.5, 95% CI 1.3 to 4.8, P = 0.005), being a second or subsequent PIVC to be placed during hospitalisation (OR 2.2, 95% CI 1.2 to 4.0, P = 0.007), flush frequency (P = 0.02) with “other” flushing frequency having increased odds of 7.4 (95% CI 1.6 to 35.0, P = 0.01) compared to flushing every 1 to 4 hours, and PIVC flush solution (P = 0.019) with flushing with compound sodium lactate solution having increased odds of 7.3 (95% CI 1.7 to 30.3, P = 0.007).

**Table 5:** Univariable binary regression analysis of factors associated with peripheral intravenous catheter (PIVC) complications

Variable	Category	Number	Odds ratio	95% CI	Category P‐value	Variable P‐value
Species	Cat	56	Base			0.81
	Dog	307	1.1	0.6 to 2.1	0.81	
Patient appointment type	Routine	187	Base			0.13
	Emergency	176	1.4	0.9 to 2.3	0.13	
Personnel role	RVN	259	Base			0.19
	Veterinary surgeon	83	1.1	0.6 to 1.8	0.84	
	Student	21	0.3	0.1 to 1.1	0.07	
Hand hygiene	No	119	Base			0.25
	Yes	214	0.7	0.5 to 1.2	0.25	
	Missing	30				
Patient demeanour	Calm and relaxed, minimal restraint required	219	Base			0.51
	Agitated, nervous, restless, required tight restraint for placement	98	0.7	0.4 to 1.2	0.22	
	Exhibiting signs of aggressive behaviour, required tight restraint for placement	15	1.2	0.4 to 3.5	0.80	
	Unable to place PIVC without chemical restraint	24	0.6	0.2 to 1.7	0.35	
	Missing	7				
Location of patient	Floor	162	Base			0.47
	Table	193	1.2	0.7 to 1.9	0.47	
	Missing	8				
Number of attempts to place PIVC	1	299	Base			0.003
	>1	64	2.4	1.4 to 4.1	0.003	
PIVC number since admission	1	281	Base			<0.001
	>1	82	2.5	1.5 to 4.3	<0.001	
Skin disinfection	Alcohol only	62	Base			0.64
	Alcohol + chlorhexidine	300	0.9	0.5 to 1.6	0.64	
	Missing	1				
Local anaesthetics used	No	268	Base			0.76
	Yes	67	0.9	0.5 to 1.7	0.76	
	Missing	28				
PIVC material	Polyurethane	77	Base			0.35
	Teflon	285	0.8	0.4 to 1.3	0.35	
	Missing	1				
Catheter size	18	14	Base			0.39
	20	173	0.9	0.3 to 2.9	0.80	
	22	165	1.1	0.3 to 3.5	0.93	
	24	10	2.5	0.5 to 13.6	0.29	
	Missing	1				
PIVC Limb	Thoracic	338	Base			0.16
	Pelvic	19	2.0	0.8 to 5.1	0.16	
	Missing	6				
Connector onto PIVC	T‐port	213	Base			0.37
	Y‐connector	113	0.8	0.5 to 1.4	0.51	
	Other	35	1.5	0.7 to 3.2	0.28	
	Missing	2				
Was a needle free system used	Yes	290	Base			0.47
	No	53	1.3	0.7 to 2.4	0.47	
	Missing	20				
Tape used to secure PIVC	Durapore	134	Base			0.42
	Elastic fabric tape	184	0.8	0.5 to 1.3	0.40	
	Other	43	1.3	0.6 to 2.6	0.54	
	Missing	2				
Blood collected via PIVC	No	251	Base			0.36
	Yes	109	1.3	0.8 to 2.1	0.36	
	Missing	3				
Flush frequency	1 to 4 hours	162	Base			0.03
	5 to 11 hours	84	1.2	0.7 to 2.2	0.56	
	12 to 24 hours	65	1.9	1.0 to 3.5	0.05	
	Other	9	6.8	1.6 to 28.3	0.009	
	Missing	43				
Flush solution type	0.9% sodium chloride	293	Base			0.02
	Heparin saline	22	1.6	0.7 to 4.0	0.29	
	Compound sodium lactate	10	6.7	1.7 to 26.4	0.007	
	Missing	38				
Intravenous glucocorticoids administered	No	343	Base			0.32
	Yes	17	0.5	0.1 to 21.9	0.32	
	Missing	3				
Intravenous anti‐emetic medication administered	No	263	Base			0.15
	Yes	97	1.5	0.9 to 2.4	0.15	
	Missing	3				
Intravenous analgesia (non‐steroidal anti‐inflammatory drugs and opioids)	No	97	Base			0.90
	Yes	263	01.0	0.6 to 1.7	0.90	
	Missing	3				

RVN Registered veterinary nurse

**Table 6:** Final model of the multivariable binary regression analysis of factors associated with a peripheral intravenous catheter (PIVC) complication

Variable	Category	Number	Unadjusted odds ratio	95% confidence interval	Category P‐value	Variable P‐value
Number of attempts to place PIVC	1	265	Base			0.005
	>1	55	2.5	1.3 to 4.8	0.005	
PIVC number since hospitalisation	1	243	Base			0.007
	>1	77	2.2	1.2 to 4.0	0.007	
PIVC flush frequency	1 to 4 hours	162	Base			0.02
	5 to 11 hours	81	1.0	0.5 to 1.9	0.90	
	12 to 24 hours	65	2.5	1.0 to 3.8	0.4	
	Other	9	6.5	1.4 to 29.6	0.02	
PIVC flush solution	0.9% sodium chloride	289	Base			0.019
	Heparin saline	18	1.7	0.6 to 4.8	0.35	
	Compound sodium lactate	10	7.3	1.7 to 30.3	0.007	

## DISCUSSION

This study describes PIVC placement, use and complications within multiple veterinary institutes in the UK. The finding that 26.7% PIVCs were associated with a complication aligns with other human and veterinary reports but is nonetheless concerning that this extensively used device causes problems in so many patients. The most common complications reported in our study were limb swelling and/or suspected phlebitis and PIVC dislodgement and/or patient interference, occurring in 11.5% and 7.9% of PIVCs, respectively. Other veterinary studies have reported phlebitis, line breakage and extravasation to be the most common observable complication occurring in 3.5% to 7% of PIVCs (Bush et al., 2020; Simpson & Zersen, 2022). Phlebitis may be mechanical due to physical damage and friction caused by the PIVC, chemical caused by infusion of irritant solutions, or infectious caused by bacterial colonisation of the PIVC (Higginson & Parry, 2011). A meta‐analysis of the literature describing PIVC‐induced phlebitis in humans reported that neither PIVC size nor PIVC duration in‐situ were associated with risk of phlebitis, which align with the findings of our study (Chang & Peng, 2018). PIVC complications resulted in premature removal of 21% of the PIVCs placed in our study, which is lower than 46% of PIVCs requiring premature removal reported in another veterinary study (Crisi et al., 2022). Possible explanations for this difference include different placement and management techniques, and that we did not request a standard frequency of PIVC insertion site assessment, whilst PIVC insertion sites were assessed every 12 hours in the study by Crisi et al., 2022. Further research needs to be undertaken which aims to identify interventions which can decrease the frequency of PIVC complications, and define the optimal time interval for assessment of potential PIVC complications.

PIVCs which required more than one attempt to be placed had increased odds of 2.5 for a complication compared to those which were placed at first attempt. Possible explanations for this including vein damage during a failed attempt which may increase the risk for extravasation or thrombosis, or cause discomfort for the patient who may be more likely to then interfere with the PIVC. The first‐time placement success of PIVCs of 82% in this study parallels success rates from human studies (Carr et al., 2019; Marsh, Webster, Larsen, Genzel, et al., 2018). A feature that human and veterinary medicine share is that PIVCs are predominantly placed by nurses or technicians, with 72% of PIVCs being placed by RVNs in our study and 71% of PIVCs being placed by nurses in human medicine (Alexandrou et al., 2018). Skilled professionals who have undertaken additional training in vascular access, known as vascular access specialists, have been shown to have higher first‐time placement rates, shorter time for placement and lower complication rates in human medicine (Marsh, Webster, Larsen, Genzel, et al., 2018). The finding of our study that 17.5% of PIVCs required more than one attempt at placement and this was associated with a higher risk of complication suggests there is rationale for ongoing training in vascular access and development of devices which could improve first placement success rates.

PIVC flushing frequency and flush fluid type were associated with odds of a complication. Flushing frequency in the “other” group contained PIVCs that were not flushed because of concurrent administration of intravenous fluid therapy, PIVCs which were flushed only after intravenous medication was administered, PIVCs which were flushed “as needed” or had inconsistent time intervals between flushing. Studies in humans assessing flush frequency and PIVC complications have reported no difference in outcomes when a flush frequency of every 6 hours was compared to every 24 hours, and when every 12 hours was compared to every 24 hours (Keogh et al., 2016; Schreiber et al., 2015). Our recommendation is to routinely flush PIVCs at least every 24 hours with a consistent time interval between flushings. Veterinary studies have reported similar outcomes when 0.9% sodium chloride is used compared to heparinised sodium chloride to maintain the patency of peripheral and central venous, and arterial catheters (Sasaki et al., 2020; Silver et al., 2017; Ueda et al., 2013; Vose et al., 2019). Given those findings, and the results of our study that show using compound sodium lactate solution had increased odds of having a PIVC complication, flushing with either 0.9% sodium chloride or heparin saline is recommended, and compound sodium lactate solution should not be used.

An increased likelihood of a PIVC complication was identified in PIVCs which were a second or subsequent PIVC being placed during a patient's hospitalisation. There are numerous possible explanations for this finding, including patients requiring a second PIVC did so because of a complication with their first PIVC, patients were hospitalised for longer which has been reported as a risk factor in veterinary studies or there could be differences in the placement or management of second or subsequent PIVCs that were not identified in our questionnaire. (Granger et al., 2023; Simpson & Zersen, 2023). As this data was not collected in our study, conclusions regarding the reason why second or subsequent PIVCs had increased odds of a complication cannot but drawn. A prospective study that allows the inclusion of multiple catheters per patient may help elucidate whether patient or personnel factors contribute to the increased risk of complication with second or subsequent catheters.

Hand hygiene was not performed prior to placement in approximately one of three of PIVCs in this study, with veterinarians being the group least commonly performing hand hygiene. Poor compliance with hand hygiene at the time of PIVC placement has also been reported in human studies (Hirschmann et al., 2001; Zingg et al., 2009). Hand hygiene is a component of most PIVC care bundles in human medicine, and should be performed prior to each interaction with a PIVC as appropriate hand hygiene has been shown to decrease PIVC complications in humans (Hirschmann et al., 2001; Loveday et al., 2014; Ray‐Barruel et al., 2019). Although hand hygiene was not identified as a factor associated with complications in the univariable analysis in our study, we did not investigate hand hygiene throughout the time the PIVC was in situ and this could be incorporated into future studies assessing factors associated with PIVC complications. Nonetheless, the results of our study serve as an important reminder that veterinary professions have significant room for improvement of hand hygiene.

In our study, 18.6% of PIVCs were placed with alcohol skin cleaning only. Methylated spirit has been documented to reduce skin bacterial colony forming units by only 68% compared to 100% reduction when using 4% chlorhexidine gluconate, with 4% chlorhexidine gluconate providing sustained reduction of skin bacterial colonisation (Coolman et al., 1998; Dorey‐Phillips & Murison, 2008). Appropriate skin disinfection prior to PICS placement is imperative because the most common cause of local catheter site infections has been reported to be bacterial colonisation at the PIVC insertion site (Zhang et al., 2016). The use of 2% chlorhexidine with 70% isopropyl alcohol has been associated with lower PIVC bacterial colonisation and lower frequency of regional infections than 5% povidone iodine with 69% ethanol in studies of humans (Guenezan et al., 2021; Mimoz et al., 2015). Therefore, chlorhexidine‐containing skin disinfectants are preferable to alcohol alone or povidone iodine and alcohol, despite skin disinfectant type not being associated with odds of a complication in our study. The current recommendation is 2% chlorhexidine with alcohol for skin preparation in humans, and 2% chlorhexidine should be considered the lowest concentration to use for skin preparation in cats and dogs because chlorhexidine resistance has been documented in the bacterial flora of these species (Swales & Cogan, 2017).

Despite performing the study for 12 months over 19 institutes, data for only 363 PIVCs was available for complication analysis. There was also an uneven distribution of submissions across institutes. Five institutes contributed to 64% of PIVC submission, all of which were large referral hospitals, and the standard operating procedures from these five hospitals (three of which are owned by the same corporate veterinary company) may have reduced the variation of PIVC management approaches. Almost all institutes (17 of 19) had RCVS hospital status, which represents 7% of the RCVS accredited hospitals and only 14 cases came from a mixed (farm and companion animal) veterinary practice, which represents 2% of practices registered with the RCVS who care for both farm and companion animals. Therefore, the data captured in this study may not reflect general practice behaviour, and the inclusion criteria of the study may have prevented many general practices from being able to contribute data to this study.

On reflection, it would have been beneficial to record additional parameters including patient age and total length of hospitalisation. We intentionally included only one PIVC per patient, but exploring the risk of consecutive PIVCs may also have been beneficial. Other factors which may have been valuable to record would have included PIVC securing techniques, bandaging material used, how PIVC inspections occurred (including whether banding material was routinely removed for PIVC inspection), how frequently PIVC inspection occurred and hand hygiene performed during each interaction with the PIVC. We were mindful that the questionnaire had to be succinct whilst also capturing the data which was deemed to be relevant to PIVC complication based on the literature at the time of development and experience of the authors. Any second iteration, if used, would aim to capture this additional data. The lack of standardisation of patient preparation, PIVC placement and management allowed us to simultaneously explore numerous factors which could be associated with PIVC complications. However, there are differences which are likely to exist between institutes which could influence the results of this study such as how frequently PIVCs were being assessed for a complication, variations between individuals when they interact with a PIVC such as forcefulness of PIVC flushing and how effectively an injection port is cleaned in addition to taping and bandaging techniques. All these variables could contribute to likelihood of PIVC complication. Therefore, the factors associated with risk of complication identified in this study may not transfer to risks of PIVC complications in institutes which manage their PIVCs differently or manage a different patient cohort to the institutes enrolled in this study. A future study could implement a standard operating procedure or PIVC care bundle across multiple institutes in an attempt to decrease the variability of PIVC management between institutes, and could compare those results to the previous PIVC management strategy in an audit‐reaudit analysis.

Although PIVCs are used daily in veterinary hospitals across the world, 26.7% of PIVCs were associated with complications in this study. This study serves as a reminder that hand hygiene should be performed prior to PIVC placement, in addition to adequate skin preparation. Factors associated with increased risk for PIVC complication include being a second or subsequent PIVC placed, having more than one attempt for PIVC placement, flushing at irregular time intervals, and using compound sodium lactate for flushing the PIVC. These findings could serve as a basis for a PIVC care bundle in cats and dogs, which would then need validating by a prospective study.

### Author contributions

**E. Haskey:** Conceptualization (equal); data curation (equal); formal analysis (equal); funding acquisition (equal); investigation (equal); methodology (equal); project administration (equal); writing – original draft (equal). **V. Maund:** Conceptualization (equal); data curation (equal); formal analysis (equal); funding acquisition (equal); investigation (equal); methodology (equal); project administration (equal); writing – original draft (equal). **B. Browse:** Conceptualization (equal); data curation (equal); formal analysis (equal); funding acquisition (equal); investigation (equal); methodology (equal); project administration (equal); writing – original draft (equal). **C. Heard:** Conceptualization (equal); data curation (equal); formal analysis (equal); funding acquisition (equal); investigation (equal); methodology (equal); project administration (equal); writing – original draft (equal). **C. O'Donnell:** Conceptualization (equal); data curation (equal); formal analysis (equal); funding acquisition (equal); investigation (equal); methodology (equal); project administration (equal); writing – original draft (equal). **K. Davison:** Conceptualization (equal); data curation (equal); formal analysis (equal); funding acquisition (equal); investigation (equal); methodology (equal); project administration (equal); writing – original draft (equal). **C. Hertel:** Conceptualization (equal); data curation (equal); formal analysis (equal); funding acquisition (equal); investigation (equal); methodology (equal); project administration (equal); writing – original draft (equal). **E. Booth:** Conceptualization (equal); data curation (equal); formal analysis (equal); funding acquisition (equal); investigation (equal); methodology (equal); project administration (equal); writing – original draft (equal). **S. Lawrence:** Conceptualization (equal); data curation (equal); formal analysis (equal); funding acquisition (equal); investigation (equal); methodology (equal); project administration (equal); writing – original draft (equal). **E. Dever:** Conceptualization (equal); data curation (equal); formal analysis (equal); funding acquisition (equal); investigation (equal); methodology (equal); project administration (equal); writing – original draft (equal). **L. Bowe:** Conceptualization (equal); data curation (equal); formal analysis (equal); funding acquisition (equal); investigation (equal); methodology (equal); project administration (equal); writing – original draft (equal). **H. Taylor:** Conceptualization (equal); data curation (equal); formal analysis (equal); funding acquisition (equal); investigation (equal); methodology (equal); project administration (equal); writing – original draft (equal). **K. Hall:** Conceptualization (equal); data curation (equal); formal analysis (equal); funding acquisition (equal); investigation (equal); methodology (equal); project administration (equal); writing – original draft (equal). **K. Trimble:** Conceptualization (equal); data curation (equal); formal analysis (equal); funding acquisition (equal); investigation (equal); methodology (equal); project administration (equal); writing – original draft (equal). **M. Junior:** Conceptualization (equal); data curation (equal); formal analysis (equal); funding acquisition (equal); investigation (equal); methodology (equal); project administration (equal); writing – original draft (equal). **C. Fennell:** Conceptualization (equal); data curation (equal); formal analysis (equal); funding acquisition (equal); investigation (equal); methodology (equal); project administration (equal); writing – original draft (equal). **N. Stevenson:** Conceptualization (equal); data curation (equal); formal analysis (equal); funding acquisition (equal); investigation (equal); methodology (equal); project administration (equal); writing – original draft (equal). **A. Sterritt:** Conceptualization (equal); data curation (equal); formal analysis (equal); funding acquisition (equal); investigation (equal); methodology (equal); project administration (equal); writing – original draft (equal). **E. Penn:** Conceptualization (equal); data curation (equal); formal analysis (equal); funding acquisition (equal); investigation (equal); methodology (equal); project administration (equal); writing – original draft (equal). **L. Nowell:** Conceptualization (equal); data curation (equal); formal analysis (equal); funding acquisition (equal); investigation (equal); methodology (equal); project administration (equal); writing – original draft (equal). **A. Collins:** Conceptualization (equal); data curation (equal); formal analysis (equal); funding acquisition (equal); investigation (equal); methodology (equal); project administration (equal); writing – original draft (equal). **E. Jones:** Conceptualization (equal); data curation (equal); formal analysis (equal); funding acquisition (equal); investigation (equal); methodology (equal); project administration (equal); writing – original draft (equal). **C. Scudder:** Conceptualization (equal); data curation (equal); formal analysis (equal); funding acquisition (equal); investigation (equal); methodology (equal); project administration (equal); writing – original draft (equal).

### Conflict of interest

None of the authors of this article has a financial or personal relationship with other people or organisations that could inappropriately influence or bias the content of the paper.

## Supporting information


**Data S1.** The questionnaire used to collect the study data.
**Table S1.** Additional information regarding PIVC placement and maintenance.

## Data Availability

The data that support the findings of this study are openly available in Figshare.com at https://doi.org/10.6084/m9.figshare.25283356.v1.
