# Supplementary material for: Placement, management and complications associated with peripheral intravenous catheter use in UK small animal practice
Source: J Small Anim Pract. 2024 Sep 5;66(1):3–13. doi: 10.1111/jsap.13782 (PMC11736090; doi:10.1111/jsap.13782)
Supplement: Supplementary file 1 — Data S1. The questionnaire used to collect the study data. Table S1. Additional information regarding PIVC placement and maintenance. [file JSAP-66-3-s001.docx]

Supplement 1

The questionnaire used to collect the study data


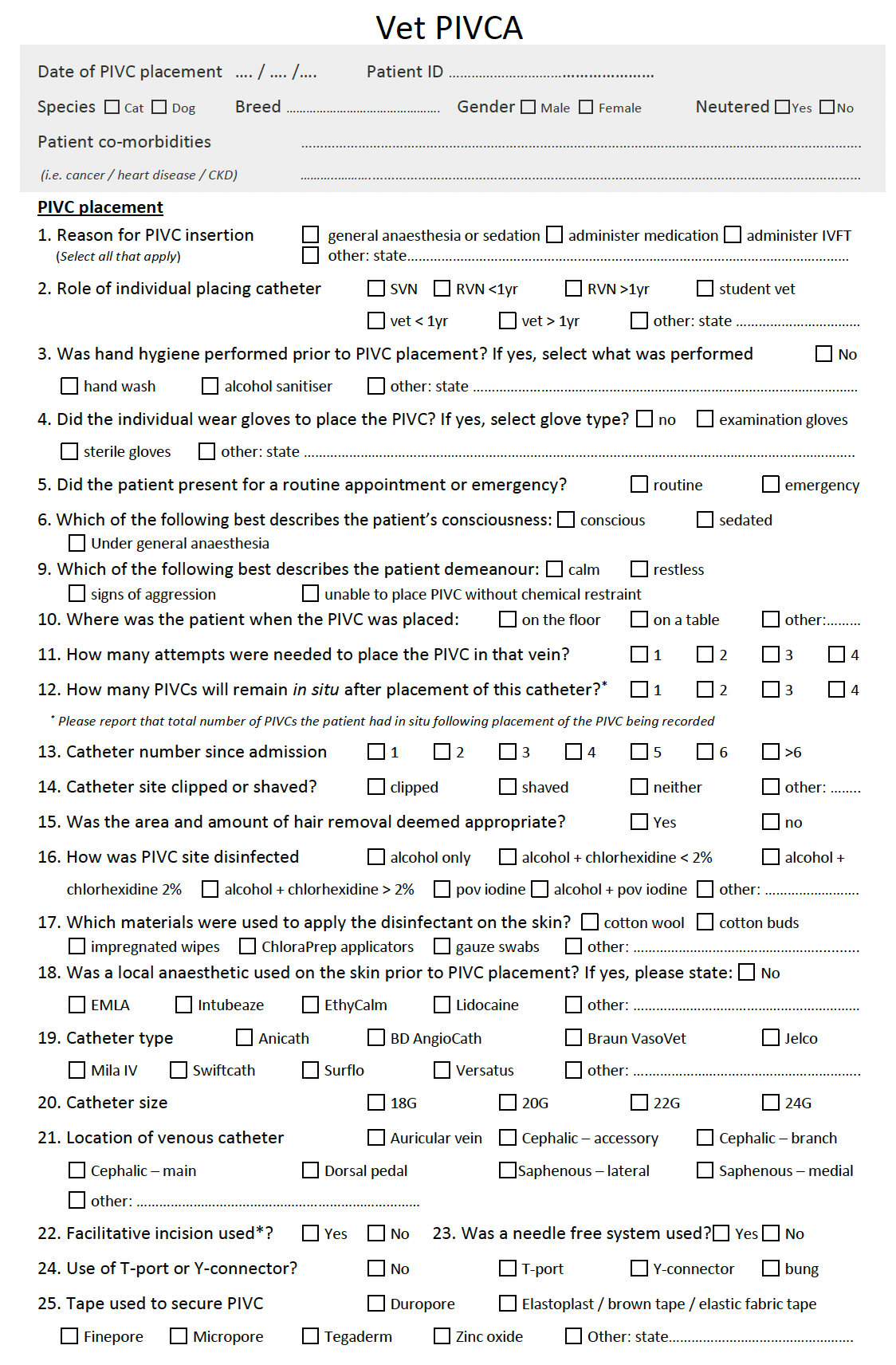


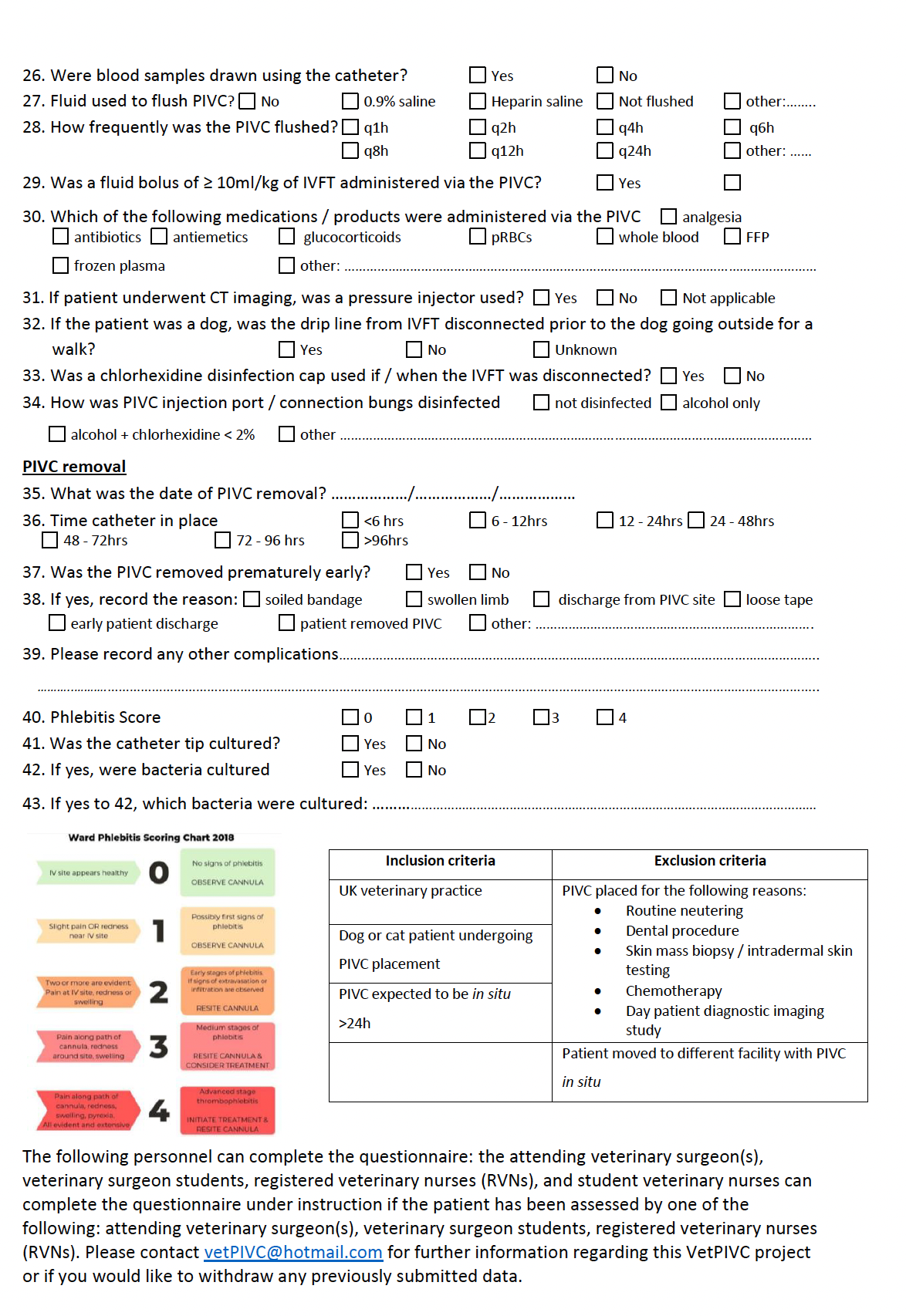


Supplementary 2

Additional information regarding PIVC placement and maintenance

| Variable | Dog | Cat |
| --- | --- | --- |
| Did the individual wear gloves to place the PIVC? If yes, select glove type | No = 270 (83.1%)  Non-sterile gloves = 55 (16.9%) | No = 48 (84.2%)  Non-sterile gloves = 8 (14.0%)  Sterile gloves = 1 (1.8%) |
| Which of the following best described the patient’s consciousness? | Conscious = 272 (83.7%)  Sedated = 44 (13.5%)  Under general anaesthesia = 5 (1.5%)  Missing = 4 (1.2%) | Conscious = 45 (78.9%)  Sedated = 12 (21.1%) |
| Which of the following best describes the patient demeanour? | Calm = 199 (61.2%)  Restless = 86 (26.5%)  Showing signs of aggression = 12 (3.7%)  Unable to place PIVC without chemical restraint = 19 (5.8%)  Missing = 9 (2.8%) | Calm = 29 (50.9%)  Restless = 16 (28.1%)  Showing signs of aggression = 4 (7.0%)  Unable to place PIVC without chemical restraint = 6 (10.5%)  Missing = 2 (3.5%) |
| Catheter site clipped or shaved? | Clipped = 268 (82.5%)  Shaved = 52 (16.0%)  Missing = 5 (1.5%) | Clipped = 53 (93.0%)  Shaved = 3 (5.3%)  Missing = 1 (1.8%) |
| What materials were used to apply the disinfectant on the skin? | Cotton wool = 79 (24.3%)  Impregnated wipes = 156 (48.0%)  ChloraPrep™ applicators = 49 (15.1%)  Gauze swab = 100 (30.8%)  Other = 2 (0.6%)  Missing = 7 (2.2%)  For 12 PIVCs both cotton wool and ChloraPrep™ applicators were used, for 45 PIVCs both gauze swabs and impregnated wipes were used, for three PIVCs both impregnanted wipes and ChloraPrep™ applicators were used, and for five PIVCs both gauze swabs and ChloraPrep™ applicators were used and for 3 dogs both impregnated swabs and ChloraPrep™ applicators were used. | Cotton wool = 9 (15.8%)  Impregnated wipes = 30 (52.6%)  ChloraPrep™ applicators = 16 (28.1%)  Gauze swab = 13 (22.8%)  For one PIVC, both cotton wool and impregnated wipes were used, for one PIVC gauze swabs and impregnated wipes were used, for three PIVCs both cotton wool and ChloraPrep™ applicators were used, for one PIVC both impregnanted wipes and ChloraPrep™ applicators were used, for four PIVCs both gauze swabs and ChloraPrep™ applicators were used, and for one PIVC both impregnated swabs and ChloraPrep™ applicators were used. |
| Catheter type | BD AngioCath™ = 2 (0.6%)  B Braun VasoVet® = 14 (4.3%)  Jelco® = 231 (71.1%)  Terumo Surflo® = 16 (4.9%)  B Braun™ Introcan® = 41 (12.6%)  Terumo Surflash® = 12 (3.7)  Other = 3 (0.9%)  Missing = 6 (1.8%) | BD AngioCath™ = 2 (3.5%)  B Braun VasoVet® = 2 (3.5%)  Jelco® = 36 (63.2%)  Terumo Surflo® = 4 (7.0%)  B Braun™ Introcan® = 12 (21.1%)  Other = 1 (1.8%) |
| Facilitative incision used | Yes = 4 (1.2%)  No = 294 (90.5%)  Missing = 27 (8.3%) | Yes = 2 (3.5%)  No = 54 (94.7%)  Missing = 1 (1.8%) |
| Blood samples drawn from the PIVC | Yes = 87 (26.8%)  No = 227 (69.8%)  Missing = 11 (3.4%) | Yes = 23 (40.4%)  No = 34 (59.6%) |
| Was a fluid bolus of ≥10ml/kg of IVFT administered via the PIVC? | Yes = 29 (8.9%)  No = 231 (71.1%)  Missing = 65 (20.0%) | Yes = 3 (5.3%)  No = 49 (86.0%)  Missing = 58 (8.8%) |
| If the patient underwent CT imaging was a pressure injector used? | Yes = 25 (7.7%)  No = 67 (20.6%)  Not applicable = 225 (69.2)  Missing = 8 (2.5%) | No = 7 (12.3%)  Not applicable = 49 (86.0%)  Missing = 1 (1.8%) |
| Was the intravenous giving set detached from the PIVC when the patient was taken for a walk? | No = 9 (2.8%)  Yes = 293 (90.2%)  Missing data = 23 (7.1%) | N/A |
| Was a chlorhexidine disinfection cap used if / when the IVFT was disconnected? | No = 226 (69.5%)  Yes = 56 (17.2%)  Missing data = 32 (9.8%) | Missing data = 57 |
| Does your institute routinely clean needle free bungs / connection ports prior to reconnection to intravenous fluid therapy or injection of medications | No = 104 (32.0%)  Yes = 209 (64.3%)  Missing data = 17 (5.2%) | No = 14 (24.6%)  Yes = 40 (70.2%)  Missing data = 3 |
| How was PIVC injection port / connection bungs disinfected? | Alcohol = 120 (36.9%)  Chlorhexidine + alcohol = 78 (24.0%)  Missing data = 120 (36.9%) | Alcohol = 22 (38.6%)  Chlorhexidine + alcohol = 18 (31.6%)  Missing data = 17 (29.8%) |
| Apart from anaesthetic agents and intravenous fluids, which medications were administered via the PIVC | Analgesics = 240 (73.8%)  Antibiotics = 141 (43.4%)  Anti-emetics = 84 (25.8%)  Steroids = 14 (4.3%)  Blood product = 2 (.6%)  Other^‡^ = 6 | Analgesics = 32 (56.1%)  Antibiotics = 15 (26.3%)  Anti-emetics = 15 (26.3%)  Steroids = 3 (5.3%)  Blood product = 2 (3.5%)  Other^‡^ = 3 |

^‡^ Other, a percentage is not provided as more than one medication may have been administered via the same catheter.
